# Mapping of Pro-Equity Interventions Proposed by Immunisation Programs in Gavi Health Systems Strengthening Grants

**DOI:** 10.3390/vaccines11020341

**Published:** 2023-02-02

**Authors:** Joelle Ducharme, Gustavo Caetano Correa, Heidi W. Reynolds, Alyssa B. Sharkey, Virginia A. Fonner, Mira Johri

**Affiliations:** 1 Measurement, Evaluation and Learning Department, Gavi, The Vaccine Alliance, 1218 Le Grand-Saconnex, Switzerland; gcorrea@gavi.org (G.C.C.); hreynolds@gavi.org (H.W.R.); 2 School of Public and International Affairs, Princeton University, Princeton, NJ 08544, USA; asharkey@princeton.edu; 3 Global Health Population and Nutrition, FHI 360, Durham, NC 27701, USA; gfonner@fhi360.org; 4 Carrefour de l’Innovation, Centre de Recherche de l’Université de Montréal (CRCHUM), Montréal, QC H2X 0A9, Canada; mira.johri@umontreal.ca; 5 Département de Gestion, D’évaluation, et de Politique de Santé, École de Santé Publique de l’Université de Montréal (ESPUM), Montréal, QC H3N 1X9, Canada

**Keywords:** immunization, vaccines, zero-dose, equity, health systems

## Abstract

Reaching zero-dose (ZD) children, operationally defined as children who have not received a first dose of the diphtheria, tetanus, and pertussis (DTP1) vaccine, is crucial to increase equitable immunisation coverage and access to primary health care. However, little is known about the approaches already taken by countries to improve immunisation equity. We reviewed all Health System Strengthening (HSS) proposals submitted by Gavi-supported countries from 2014 to 2021 inclusively and extracted information on interventions favouring equity. Pro-equity interventions were mapped to an analytical framework representing Gavi 5.0 programmatic guidance on reaching ZD children and missed communities. Data from keyword searches and manual screening were extracted into an Excel database. Open format responses were analysed using inductive and deductive thematic coding. Data analysis was conducted using Excel and R. Of the 56 proposals included, 51 (91%) included at least one pro-equity intervention. The most common interventions were conducting outreach sessions, tailoring the location of service delivery, and partnerships. Many proposals had “bundles” of interventions, most often involving outreach, microplanning and community-level education activities. Nearly half prioritised remote-rural areas and only 30% addressed gender-related barriers to immunisation. The findings can help identify specific interventions on which to focus future evidence syntheses, case studies and implementation research and inform discussions on what may or may not need to change to better reach ZD children and missed communities moving forward.

## 1. Introduction

While global immunisation coverage has improved monumentally since the 1980s, progress in increasing coverage for different antigens has slowed or stalled over the last several years and has even declined during the COVID-19 pandemic [1,2]. Of particular concern are children who have not received any routine vaccination, referred to as zero-dose (ZD) children. These children are defined operationally by the Immunization Agenda 2030 (IA2030) as those who have not received a first dose of the diphtheria, tetanus, and pertussis (DTP1) vaccine in their first year of life [3]. With the COVID-19 pandemic straining health systems and severely impacting routine immunisation services in many countries, the number of ZD children—estimated at 14 million in 2019—increased by 34% globally in 2021, when there were approximately 18.2 million ZD children [4]. Of these, 12.5 million (68%) lived in one of the 57 Gavi-supported countries^1^ [4]. Gavi-supported countries are countries that are eligible to apply for Gavi support, determined by their national income. In 2020, countries with a Gross National Income (GNI) per capita equal to or less than US $1630 over the last three years were eligible for support [5]. It is thus now more critical than ever to reverse this trend and reach the remaining unimmunised children. Reaching ZD children and missed communities, which are operationally defined as being home to clusters of ZD and under-immunized children, is a central component of both IA2030 and Gavi, the Vaccine Alliance’s strategic plan for 2021–2025 (Gavi 5.0), with a vision of “leaving no one behind with immunisation” [3,6]. Reaching these populations is expected to bring more children to full immunisation and increase access to primary health care [7,8]. This, in turn, is an important component towards achieving universal health coverage, especially in low- and middle-income countries (LMICs) [9].

Even though the concept of equity has been guiding global immunisation efforts and was already a key principle of the Gavi 4.0 strategy (2016–2020), the stalled progress in recent years and backsliding of routine immunisation during the pandemic have highlighted the inability of traditional interventions to maintain high coverage and reach the approximately 15% of children worldwide who remain unvaccinated with DTP1 [2]. Adding to the complexity is that ZD children are among the world’s most vulnerable populations and are believed to face complex and overlapping deprivations, with several proximal, distal and greater contextual determinants (e.g., gender, conflict, and health systems factors) at play. Indeed, they often have a lower socioeconomic status, belong to religious or ethnic minorities, suffer from childhood malnutrition and are children of mothers with lower levels of education and empowerment, making them more socially and economically disadvantaged [9,10,11,12,13]. Along these lines, the Equity Reference Group for Immunisation (ERG) has emphasized and called for a greater focus on four key areas to reach ZD children, namely conflict-affected, urban poor, and remote rural areas as well as gender-related barriers. While those overlapping vulnerabilities make it particularly challenging to reach ZD children with vaccination, the potential benefits to children’s’ health and development, to their families and communities, and to societies, are immense. 

While the concept of “zero-dose” children has existed for several years [14], it only recently came to the forefront of the attention of the global health community and there is still an important lack of knowledge about which interventions can be implemented to best reach them, how much they cost, and how to sustain progress. Understanding which interventions are already being implemented is one step towards building this evidence base. To the best of our knowledge, only one study has been published that explores this question. Dadari and colleagues mapped pro-equity strategies being implemented in thirteen Gavi-supported countries through review of Joint Appraisal (JA) reports from 2016 to 2019 [15]. JA reports are documents submitted to Gavi by countries every year detailing all Gavi-supported activities implemented, progress and performance. The authors found that all thirteen countries had in place over 250 interventions aiming to increase vaccine equity and concluded that further efforts should be made to do a similar mapping with other types of Gavi documents and across more countries to establish a more complete picture [15].

Building on this foundation, the current paper presents findings from a similar mapping in Gavi Health System Strengthening (HSS) proposals. These documents are particularly relevant because they are focused on achieving equitable immunisation coverage and have by far the largest envelope of all Gavi cash grants [16]. Indeed, as of December 2021, commitments for HSS support from 2000 to 2025 for all Gavi-supported countries amounted to US $3005.4 million. In comparison, the next biggest funding lines were operational support with an envelope of US $935.5 m and immunisation services support with US $355.9 m [17]. In HSS proposals for the Gavi 4.0 strategy period, countries had to articulate how funds would be used and detail clear strategies to improve immunisation access and equity, though not specifically for zero-dose children since this only became a focus in Gavi 5.0. Proposals submitted to Gavi are reviewed and approved by an Independent Review Committee (IRC) comprising experts in different fields including public health, epidemiology, and economics. We reviewed these HSS proposals to document and learn more about the pro-equity interventions used by countries to improve immunisation equity and to understand how these investments can help us prioritise interventions moving forward. 

## 2. Materials and Methods

### 2.1. Data Sources

All HSS proposals submitted by Gavi-supported countries from 2014 to 2021 inclusively were reviewed. The overall number of Gavi-supported countries during this period varied from 73 in 2014 to 57 in 2021 [5]. These proposals were most likely to have been developed and/or implemented during the Gavi 4.0 strategy period (2016–2020). Older proposals were excluded because they were written well before Gavi 4.0 and thus would likely not be focused on increasing equity. Two countries submitted two different HSS proposals during this period. In these cases, we included only the oldest one since 2014 in the analysis as those were more likely to have followed Gavi 4.0 recommendations and to have been implemented during that period. The documents were available through the “country documents” web pages on the Gavi website [18]. The final mapping included 56 HSS proposals from 55 countries (with two separate proposals for Syria related to different regions).

### 2.2. Analytical Framework Development

To collect relevant and useful information from the mapping, we developed an analytical framework based on the Gavi programmatic guidance [19] and the UNICEF mapping of JA reports [15]. Table 1 provides a list of the variables used to summarise the pro-equity strategies planned by each country to reach ZD/under-immunised children. Adapting the definition used by Dadari and colleagues [15], we defined a pro-equity intervention as any tailored or targeted approach designed to reach underserved/vulnerable populations or communities with immunisation. All other interventions planned to be implemented throughout the country or not targeted at the priority groups or areas identified as being most vulnerable were not included. We created categories of interventions by thematic areas (grouping interventions that were similar and had the same purpose) to analyse which types of pro-equity interventions countries planned to implement. We validated the categories against those used by UNICEF and the Gavi programmatic guidance to ensure they were comprehensive and aligned. When a thematic area was identified that did not fit in any of the existing categories, it was brought to an internal working group to determine whether to create new categories. A complete list of intervention categories and their definitions can be found in Table A1. 

### 2.3. Searching and Data Extraction

The search strategy consisted of a manual screening of specific sections of the HSS proposals and keyword searches. Data on interventions planned to reach the target populations were usually found in the “Objectives of the proposal” and/or “Description of Activities’’ sections. Different keywords were used to answer specific questions, such as “sustainability” and “gender” to identify whether those topics were addressed, for example. Relevant information was extracted into an Excel database, in which each row represented a different proposal and each column a variable of the analytical framework (see Table 1 for the variables included). One researcher extracted these data from all HSS proposals during February and March 2022. To run the correlation analysis in R, we also created a separate database listing all the pro-equity intervention categories included in the proposals. 

### 2.4. Data Analysis

The quantitative analysis consisted of descriptive statistics (counts, proportions, and frequencies) and was conducted on Microsoft Excel PivotTables, version 2206. Furthermore, we performed a correlation matrix using the R expand function to find the correlations of interventions in each country. This was conducted to find which interventions are often planned to be implemented together in the countries. Lastly, we performed inductive and deductive thematic coding based on information and observations noted throughout data extraction for open-ended variables. The coding was conducted by constructing a matrix in Excel. 

## 3. Results

**Figure 1:**
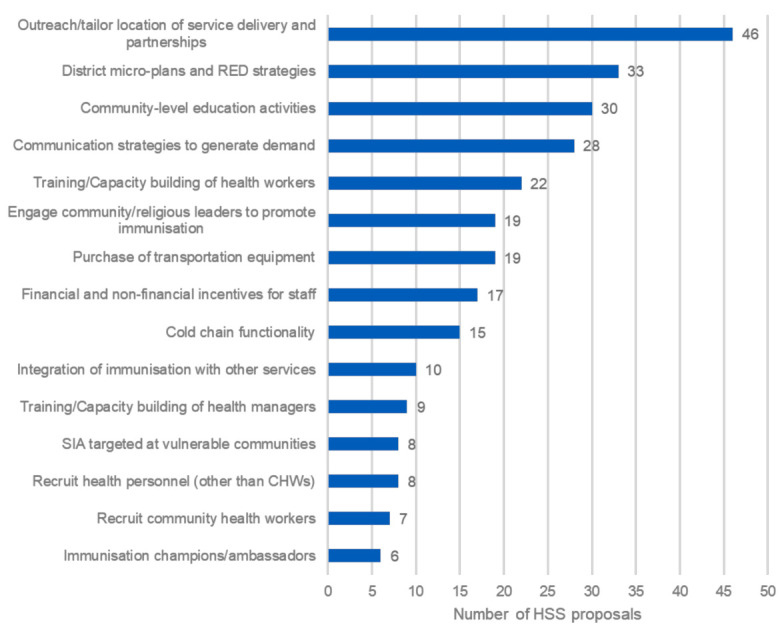
The 15 most common pro-equity intervention categories listed in the HSS proposals.

Overall, 51 of the 56 HSS proposals reviewed (91%) included at least one pro-equity intervention. When ranked by frequency (Figure 1), we found that the 15 most common pro-equity intervention categories included a mix of supply-oriented, demand-oriented and multifaceted strategies (see Table A1 for a complete list of categories and their respective definitions). We found the most common category was “outreach/tailor location of service delivery and partnerships”, with 46/56 proposals reviewed (82%) planning to implement this. This category included any activities that sought to increase immunisation coverage by either conducting outreach services or tailoring the location of service delivery to reach underserved populations. It also included mobile vaccination efforts, building new infrastructure, and creating or leveraging partnerships (e.g., with non-governmental organisations, private sector) to expand vaccine access. The second most common category was the development of microplans at the health facility or district level and/or the implementation of other “Reach Every District” (RED) strategies [20]. Microplans and RED strategies were often implemented together, but if proposals planned just one of the two, it was still included in this category. 

Three of the top six most common categories aimed to generate demand at community-level (community-level education activities, communication strategies to generate demand and engaging community and/or religious leaders to promote immunisation). On the supply-side, several countries invested in transportation (motorcycles, boats, etc.) and cold chain equipment for their most hard-to-reach districts.

Next, we looked at the proportion of HSS proposals addressing the close-ended variables of the analytical framework (Yes or No response type) as shown in Table 2. Of note, civil society organisations (CSOs) were often mentioned as key partners in implementing activities and reaching vulnerable populations. This was most often performed through implementing sensitisation and social mobilisation activities at community-level to generate demand.

Interventions addressing demand-side barriers included, for example, tailored immunisation sensitisation activities to different groups (women, religious, etc.) and conducting information, education, and communication (IEC) sessions in priority districts with communities, including educational chats, film showings, and outdoor theatres. Interventions addressing supply-side barriers, on the other hand, included conducting outreach and mobile sessions, investing in targeted infrastructure and procuring motorcycles, boats and other transportation equipment for health workers to access hard-to-reach areas.

Less than one third of the proposals (17/56) explicitly addressed gender-related barriers. Of those, sixteen included gender-responsive interventions and five proposed gender-transformative ones. Considering Gavi’s gender policy, gender-responsive approaches “adopt a gender lens to consider individual needs of different gender identities without necessarily changing the larger contextual issues that lie at the root of the gender inequities and inequalities” [21]. For example, employing female health workers may facilitate enhanced immunisation service acceptance and uptake, but would not address the underlying cultural barrier that prevents female caregivers from seeking immunisation services from male health workers. Gender-transformative approaches, on the other hand, “attempt to re-define and change existing gender roles, norms, attitudes, and practices. These interventions tackle the root causes of gender inequity and inequality and reshape unequal power relations” [21]. For example, one country planned to have community health workers promote a gender approach with the involvement of fathers for the vaccination of children in households and another sought involvement of national and local leaders to promote, advocate immunisation and serve as ‘role models’ to help increase male participation.

Regarding geographic areas of focus, namely ERG settings, twenty-seven proposals selected remote-rural areas (including hard-to-reach areas) as a priority whereas only four prioritized urban poor areas (though twenty in total selected “urban”) and four selected conflict-affected areas. 

**Figure 2:**
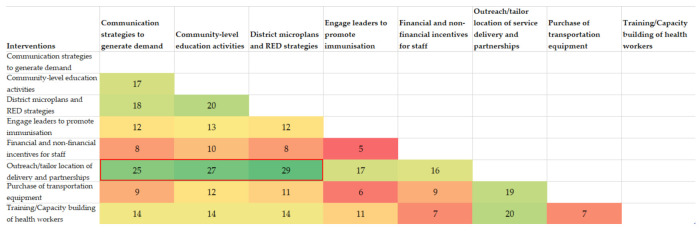
Correlation matrix representing the number of times the eight most common intervention categories were planned with each other in HSS proposals.

**Figure A1:**
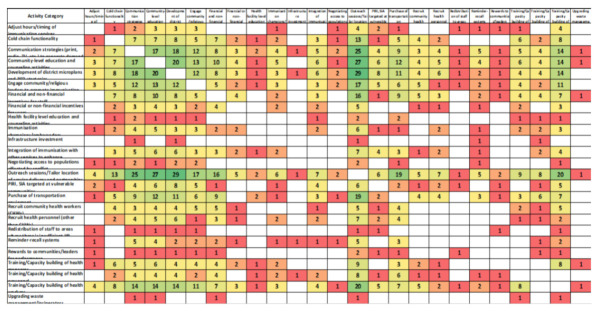
Complete correlation matrix (including all intervention categories).

The correlation matrix of different interventions in each country showed that “outreach/tailor location of service delivery and partnerships” was very strongly correlated with other interventions. Indeed, this type of intervention was planned along with developing district microplans/RED strategies in 29 HSS proposals, with community-level education activities in 27 proposals and with communication strategies to generate demand in 25 proposals (Figure 2). Thus, this suggests that outreach sessions in countries were often planned along with microplanning and community engagement activities as a “bundle” of interventions. Figure 2 depicts the correlations between the eight most common categories and the complete correlation matrix can be found in Figure A1.

We additionally found that the theory, or rationale, behind the selection of specific pro-equity interventions in the HSS proposals was often not provided. When it was provided, it was generally for unique interventions that were not commonly used by countries, such as immunisation ambassadors programs and the tool “My Village My Home” implemented in a few countries, for example. Lastly, even though most countries included some pro-equity interventions, many activities listed in the HSS proposals overall were not targeted at the priority groups or areas identified as being most vulnerable but were instead planned to be implemented at the national level or in the other, non-priority areas. These were not included in the database, nor the analysis presented here.

## 4. Discussion

Our mapping confirmed that most countries have already been proposing pro-equity interventions using Gavi HSS funds for several years and is consistent with the results from UNICEF’s mapping of JA reports [15]. It is the first time we are aware that such an analysis of pro-equity interventions in HSS proposals from all Gavi-supported countries was conducted. Importantly, we found that outreach and tailoring location of service delivery was very commonly presented in those proposals. This aligns with the observation that one of the main bottlenecks to getting children immunised reported in the proposals was the long distances to health facilities. This bottleneck is also well documented in the literature as a significant barrier to access to immunisation services [22]. Countries mostly addressed this bottleneck by conducting outreach and mobile sessions in hard-to-reach areas or areas with no health facility nearby. However, sustainability concerns and cost-effectiveness of conducting outreach and mobile sessions as compared to other long-term strategies were rarely discussed in the HSS proposals. One explanation for this might be the fact that outreach and mobile sessions have been used for a long time and are generally considered necessary to increase immunisation coverage in those contexts.

District microplans and RED strategies were also very common and were assembled into one category because they were frequently planned to be implemented concurrently and since microplanning is often the only component of RED that is implemented. The RED approach was developed by the World Health Organization (WHO), UNICEF and other partners in the Gavi Alliance to improve immunisation coverage and it includes five operational components aimed at improving vaccination coverage: re-establishment of regular outreach services, supportive supervision, on-site training, community links with service delivery, monitoring and use of data for action, and better planning and management of human and financial resources [20]. These components thus have a large span and since they were grouped together under “RED strategies” in the proposals, we could not have a clear vision on what was planned exactly in each country. It was interesting to see, however, how widespread this overall approach has become as a strategy to improve immunisation coverage and increase equity.

Interestingly, two categories were reported in the UNICEF pro-equity mapping of JA reports that were, however, not found in the HSS proposals. These were “peer support group for health providers” and “security to allow immunisation services to happen safely”.

Additionally, the results showed that several proposals focused on reaching remote-rural and hard-to-reach areas, but few prioritised the other ERG settings, namely urban poor and conflict-affected areas. To note that only proposals that explicitly stated they would prioritise those areas and developed key interventions to improve coverage there were included in the results. It is possible that other planned pro-equity interventions would address barriers in those areas, but were not clearly acknowledged as doing so. These results were not surprising, however, as we analysed HSS proposals submitted starting from 2014, while the ERG priority areas were only defined in 2018. Furthermore, recent evidence shows that unlike what we might have expected, less than 50% of ZD children live in ERG settings worldwide, suggesting that although they are key areas for prioritisation, it is unlikely that we will make considerable progress by solely targeting those settings [23]. Still, many proposals did mention having large pockets of unimmunised children in large urban areas and slums. It is estimated that 28% of un- or under-vaccinated children lived in urban and peri-urban areas and up to 15% lived in conflict-affected areas in 2020 [24]. It would thus be beneficial to pay special attention to those populations. In this sense, it is worth noting renewed efforts to reach conflict-affected populations with the launch of the Zero-Dose Immunisation Programme (ZIP) in June 2022. This initiative led by Gavi in partnership with the International Rescue Committee and World Vision aims to identify and reach ZD children in the Horn of Africa and the Sahel regions, prioritising children living in conflict settings, mobile populations, and cross-border refugees [25].

Interestingly, the results of the correlation analysis suggest that “bundles” of interventions are commonly used at the country-level as part of the strategy to increase immunisation coverage in the priority areas. Indeed, we found that outreach sessions and tailoring location of immunisation services was often implemented along with developing district microplans/RED strategies, community-level education activities as well as communication strategies to generate demand. Conversely, the analysis revealed how often certain interventions were not bundled, potentially limiting their sustainability and effectiveness. This links to increasing evidence that there are no silver bullets but rather bundles, or packages, of evidence-based interventions tailored to local context that are needed to increase immunisation coverage [26,27]. Learning efforts exploring these bundles of interventions to better reach ZD children and how to use the interventions synergistically to build off one another would be worth exploring. Developing a theory of change, among other things, would be a useful exercise to justify the bundling of activities and validate their effectiveness through implementation research or other approaches [28].

The finding that the rationale, or theory, behind the selection of specific pro-equity interventions was seldom provided in the proposals does not suggest that there is no rationale, but only that it was not clearly formulated. This made it difficult to assess the relevance and intended effects of interventions in different contexts. The few instances when a rationale was presented were generally for interventions that were not commonly found in other countries’ HSS proposals. One might reasonably assume that there was less established evidence supporting the implementation of these rarer interventions, thus the need to justify them in the proposals. Documenting the assumptions and reasoning for specific interventions, especially less common ones, would be beneficial to monitor and measure their effectiveness. For example, by developing a theory of change or a strong logical model based on evidence of good results from other similar programs. 

Furthermore, in the case of bundles, theories of change would be helpful to articulate how different interventions are expected to work synergistically to produce change. It would also help with the monitoring and evaluation of programmes. Considering this, it would be highly useful to build an evidence base of interventions, namely through implementation research, that may be used in those bundles. This has been conducted, for example, in the field of family planning, where over sixty organisations have endorsed and participated in the development, dissemination and implementation of a repository of evidence-based interventions coined “High Impact Practices”, or HIPs [29]. The group explores practices that have demonstrated impact and generate evidence around replicability, scalability, sustainability, and cost-effectiveness of the different interventions and disseminate information namely through evidence briefs. Building similar evidence on interventions aiming to reach ZD children would be extremely valuable and would help prioritisation and strategic planning for future investments. This work could also be used for advocacy, design and implementation of programs, development of policies and guidelines, and identify knowledge gaps for future research. 

Furthermore, several of the interventions listed in the proposals were not considered pro-equity according to our definition (and were thus not included in the database nor analysis), but they could easily become so if they were targeted or tailored to specific populations. For example, social mobilisation activities to generate demand aimed at the entire population of a country through mass media could become pro-equity by adapting the messaging to specific target communities. Along the same lines, capacity building of health workers could become a pro-equity intervention if the health workers received adapted training on interpersonal relations with specific vulnerable groups such as refugees, for example, or if they served a low-performing area. In short, countries do not necessarily have to go back to the drawing board to design ‘pro-equity’ interventions but should build on existing interventions and tailor and target them to areas and/or populations with large numbers of ZD children. Accurately identifying who and where zero-dose children is evidently a critical pre-requisite to be able to do this effectively. This is not to say that innovative interventions are not needed to reach ZD children, but both strategies can be used coincidently. It is also not to say that simply targeting and tailoring an intervention to a subgroup will necessarily be effective. Understanding the context, including the different vulnerabilities and barriers faced by a particular community, as well as building human-centred designs will be critical to appropriately reach the remaining unimmunised children.

A crucial point at the centre of the ongoing work around zero-dose children is the lack of an agreed upon definition of what constitutes a “pro-equity” approach. Vega & Irwin first highlighted in 2004 that pro-equity health policy should not only consider socioeconomic status, but all other social and systemic factors that influence health [30]. Wagner more specifically referred to pro-equity approaches as promoting equity for women and girls, special education needs and “marginalized” populations [31]. In the current article, we defined pro-equity interventions as “tailored or targeted approaches towards un- or under-immunised children and missed communities”. Dadari and colleagues, for their part, defined them as “strategies designed to reach underserved children and populations” [15]. However, there is no formal definition and none of the current ones explicitly address the intersectional nature of inequities. Intersectionality, a concept first coined in African American feminist literature, describes the ways in which different inequalities are linked together and are mutually reinforcing in perpetuating discrimination and disadvantage [32]. Promoting health equity has been a priority for a long time now and much effort has gone towards it. However, the fact that inequities remain today may in part be explained by the fact that even though research shows the importance of many social determinants on health, we often take a siloed view on how to address them. Policy and action have mostly failed to recognise their intersectional nature and have instead focused extensively on addressing inequities related to socioeconomic status or on specific programs without addressing social determinants, which may generate short-term results but may not promote sustainability [33]. Stakeholders should thus reflect on what can be completed differently, such as building packages of interventions addressing different, overlapping vulnerabilities for example, that might help us bridge the gap to promote equity and reach ZD children and missed communities. Having a clear and common definition of what constitutes a pro-equity intervention would be important to avoid working in silos and to help test the effectiveness of pro-equity approaches in reaching zero-dose children. 

The limitations of this study must be acknowledged. First, the HSS proposals provided an incomplete picture of pro-equity interventions being implemented at the country-level. They were limited in scope and reflected what countries planned to do with Gavi HSS funds, but Gavi is not the only source of funds for programmes. This might have led to a loss of perspective of other sectors in the findings and analysis. Secondly, the documents analysed did not report on implemented activities, but rather on plans susceptible to change in the countries’ dynamic contexts, and thus did not necessarily reflect what truly happened in the field. Furthermore, considering the length of each proposal, a search by keyword was performed. Even though we conducted a manual screening of sections likely to contain relevant information, it is likely that some information was missed. Finally, a number of subjective assessments had to be made during data extraction to decide the category of each intervention. However, inter-rater reliability was not assessed since there was only one analyst and steps were taken to maintain objectivity and avoid bias via building on the existing codes developed by UNICEF for the JA mapping and reviewing the findings with peers.

## 5. Conclusions

The findings from this mapping provide a portfolio analysis of HSS pro-equity programming in all Gavi-supported countries and can inform discussions on what may or may not need to change to better reach ZD children and missed communities in the future. Further mapping should be conducted to provide a more complete picture of pro-equity strategies being implemented in those countries beyond interventions funded by Gavi through HSS grants. The results can also help identify specific interventions that require further attention for further evidence synthesis, case studies and implementation research to learn more about their effectiveness, feasibility, acceptability, implementation cost and sustainability, among other factors. In addition to exploring new interventions, research should be conducted to investigate how to better design and implement commonly used interventions such as the ones identified in this mapping (e.g., outreach sessions, tailoring the location of service delivery, microplanning and community-level education activities) and adjust them to better reach the ZD children that are the key priority of Gavi 5.0 and IA2030.

## Figures and Tables

**Table 1:** Variables included in the analytical framework and their corresponding response type.

Variables	Response Type
Country	Open text
HSS proposal submission year	Open text
Is there at least one pro-equity intervention in the proposal?	Yes or No response
Intervention category (ies)	Open text [list all]
Description of intervention (s)	Open text
Partners engaged	Open text
Target population	Open text
Geographic areas	Open text
Involvement of civil society organisations?	Yes or No response
Are demand-side barriers addressed?	Yes or No response
Interventions addressing demand-side barriers	Open text
Are supply-side barriers addressed?	Yes or No response
Are gender barriers addressed?	Yes or No response
Gender transformative interventions	Open text
Gender responsive interventions	Open text
Are funding strategies addressed/selected?	Yes or No response
Funding strategy details	Open text
Are supplemental immunisation activities (SIAs), including periodic intensification of routine immunization (PIRIs), discussed to reach vulnerable populations?	Yes or No response
What are key barriers and enabling factors?	Open text
Is sustainability discussed?	Yes or No response
Have other health/non-health sectors been integrated?	Yes or No response

**Table 2:** Number and proportion of HSS proposals (out of 56) which addressed the close-ended variables (Yes or No response type) of the analytical framework.

Close-Ended Variables	Number of Proposals with Answer “Yes”	Proportion of Proposals with Answer “Yes”
Is there at least 1 pro-equity intervention in the proposal?	51	91%
Involvement of civil society organisations?	47	84%
Are demand-side barriers addressed?	42	75%
Are supply-side barriers addressed?	51	91%
Are gender barriers addressed?	17	30%
Are funding strategies addressed/selected?	16	29%
Are SIAs, including PIRIs, discussed to reach vulnerable populations?	15	27%
Is sustainability discussed?	47	84%
Have other health/non-health sectors been integrated?	28	50%

## Data Availability

All data supporting reported results in this article are from HSS proposals publicly available on Gavi, the Vaccine Alliance’s website gavi.org (accessed on 20 December 2022).

## Appendix A

**Table A1:** List of all pro-equity intervention categories found in HSS proposals relating to reaching ZD/under-immunised children and corresponding definitions.

Intervention Name	Definition
Immunization promotion activities: engage community/religious leaders to promote immunization	Any activities in which the explicit goal is to increase the engagement of community and/or religion leaders to promote immunization.
Immunization promotion activities: communication strategies (print, radio, TV, etc.) to generate demand	Any activities in which the explicit goal is to increase or improve communications to reach targeted groups, including through means such as local radio, theatre, skits, and mass media channels, to generate demand. This category can also include tailoring specific messages and/or translating existing material into local languages to reach certain groups/cultures.
Immunization promotion activities: immunization champions/ambassadors	Any activities that explicitly use immunization champions or ambassadors (or a similar title) to promote immunization.
Immunization promotion activities: community-level education and counselling activities	Any activities in which the explicit goal is to conduct community-based education or counselling activities to promote immunization.
Immunization promotion activities: health facility level education and counselling activities	Any activities in which the explicit goal is to conduct facility-based education or counselling activities to promote immunization.
Training/capacity building of health workers	Training or capacity building activities focused on health workers, including those providing clinical care and direct patient interaction.
Training/capacity building of health managers	Training or capacity building activities focused on health managers, including those involved with planning, directing, and coordinating nonclinical activities within health care systems.
Financial and non-financial incentives for staff	Interventions involving the use of incentives (either financial or non-financial) for staff involved in immunization programs to increase immunization coverage.
Financial or non-financial incentives for users	Interventions involving the use of incentives (either financial or non-financial) for individuals receiving vaccinations and/or caregivers bringing in minors for vaccination. Incentives can include general improvements to the service and/or environment where immunizations occur (i.e., shaded waiting area, provision of snacks, improving service quality).
Reminder-recall systems	Interventions involving systems that either remind patients of upcoming immunization visits that are due (reminders) or are overdue (recall). Reminder and recall systems could be delivered through digital (e.g., email, text message) or non-digital (phone call, letter) means.
Adjust hours/timing of immunization services	Any activities that seek to increase immunization coverage by altering, adjusting, and/or expanding the times of a healthcare facility or other location at which immunizations are offered, such as opening on the weekends or staying open later in the evenings.
Outreach sessions/tailor location of service delivery and partnerships for service delivery	Any activities that seek to increase immunization coverage by either providing outreach services to provide immunization or tailoring the location of service delivery. This category also includes mobile vaccination efforts, building new infrastructure (e.g., building new facilities or immunization centres), and creating or leveraging partnerships (e.g., with the private sector) to expand vaccine access.
Peer support groups for health providers	Any activities that seek to establish or strengthen peer support groups among health providers to provide a forum to share ideas and offer support to improve immunization programs.
Negotiating access to populations affected by conflict	Activities seeking to negotiate physical access to populations affected by conflict by identifying and negotiating secure access with all conflict participants, including state and nonstate actors, as well as their allies.
Recruit community health workers (CHWs)	Activities seeking to increase immunization coverage by recruiting community health workers to provide support to local immunization programs.
Recruit health personnel (other than CHWs)	Interventions seeking to increase immunization coverage by recruiting health personnel besides CHWs to provide or provide support to local immunization programs.
Recruit local HCWs who represent the communities they serve	Activities seeking to increase immunization by recruiting local healthcare workers who represent the communities they serve.
Security to allow immunization services to happen safely	Interventions seeking to provide security to allow immunization services to happen safety, such as by using military escorts or embedding vaccination program workers with military contingents.
Integration of immunization with other services to enhance convenience and strengthen Universal Primary Care	Interventions seeking to integrate immunization services with other healthcare services to increase coverage by enhancing convenience and strengthening Universal Primary Care.
Cold chain functionality	Interventions seeking to improve cold chain functionality, such as by identifying performance gaps, testing new equipment, etc. This category also includes procurement of new cold chain equipment, or other interventions designed to improve the cold chain.
Rewards to communities/leaders for performance	Interventions that explicitly offer rewards to communities and/or community leaders for improvements in local immunization coverage.
Development of district microplans and RED strategies	The development of microplans (at the facility and/or district level), which consist of developing an integrated set of components prepared to support the activities performed during a health campaign. RED (Reach Every District) strategies include five operational components aimed at improving vaccination coverage: re-establishment of regular outreach services; supportive supervision: on-site training; community links with service delivery; monitoring and use of data for action; better planning and management of human and financial resources Some interventions might include both microplans and REDs, whereas some might include microplans but not REDs or vice versa. Either scenario would still qualify for this intervention category.
Purchase of transportation equipment	The purchase of transportation equipment (e.g., cars, motorcycles, boats, etc.).
PIRI, SIA targeted at vulnerable communities	Activities to support periodic intensification of routine immunization (PIRI) or disease specific supplementary immunization activities (SIAs) such as campaigns
Redistribution of staff to areas where there is insufficient HR	Intentional plans to redistribute staff to areas where there are insufficient human resources to increase the capacity to carry out immunization programs.
Upgrading waste management/incinerators	Upgrading waste management and/or incinerators to improve the ability to carry out immunization programs.
